# Infectious diarrhea in tourists staying in a resort hotel.

**DOI:** 10.3201/eid0501.990123

**Published:** 1999

**Authors:** R M Hardie, P G Wall, P Gott, M Bardhan, L R Bartlett

**Affiliations:** PHLS, Communicable Disease Surveillance Centre, London, United Kingdom. R.Hardie@cdsc.nthames.nhs.uk

## Abstract

An outbreak of infectious diarrhea with 70 laboratory-confirmed cases (58 with Giardia lamblia) and 107 probable cases occurred in U.K. tourists who stayed in a hotel in Greece. After a cluster of six cases in persons who had stayed at the hotel was reported, the Communicable Disease Surveillance Centre began active case ascertainment. This outbreak illustrates the value of an approach to surveillance that integrates routine surveillance data with active case ascertainment.


### Infectious Diarrhea in Tourists Staying in a Resort Hotel

**Rachel M. Hardie,* Patrick G. Wall,† Patricia Gott,‡ Madhu Bardhan,‡ and Christopher L.R. Bartlett***
*PHLS, Communicable Disease Surveillance Centre, London, United Kingdom; †Food Safety Authority of Ireland, Dublin, Ireland; and ‡Department of Public Health, Coventry Health Authority, Coventry, United Kingdom

An outbreak of infectious diarrhea with 70 laboratory-confirmed cases (58 with *Giardia lamblia*) and 107 probable cases occurred in U.K. tourists who stayed in a hotel in Greece. After a cluster of six cases in persons who had stayed at the hotel was reported, the Communicable Disease Surveillance Centre began active case ascertainment. This outbreak illustrates the value of an approach to surveillance that integrates routine surveillance data with active case ascertainment.

A large outbreak of infectious diarrhea in residents of the United Kingdom who became infected while staying at a hotel in Greece was investigated in the month after their return. The outbreak was first identified by the Communicable Disease Surveillance Centre (CDSC) in June 1997 when a cluster of six microbiologically confirmed cases of giardiasis (all in patients who had stayed at the same hotel in Greece) were reported by a local public health epidemiologist. The cluster was identified after a school sought advice about excluding a child with diarrhea. These six patients reported that many other hotel guests had also been ill.

British guests who arrived at the hotel between 22 May and 9 June 1997 (the period of reported illness) were included in the investigation. The names of these guests were obtained from the tour operator (who sent, through travel agents, a letter to each booking group advising them of the outbreak and asking them to contact CDSC); other local public health epidemiologists and environmental health officers; and a list of ill people compiled by one particularly concerned guest.

In July, a standard questionnaire was administered by telephone to a member of each group (with contact information available) that had booked their holiday together. If the group consisted of more than one family, attempts were made to interview one member from each family. Fact sheets about giardiasis were distributed.

Of 86 booking groups on the tour operator's list, 51 groups (59%) (239 persons) were contacted and interviewed; 35 groups (41%) (138 persons) could not be contacted. Those contacted resided throughout the United Kingdom; 128 (54%) were male, and 132 (55%) were 16 years or older. Of the 239, 224 (94%) were ill while on holiday or within a few days of return; diarrhea was more commonly reported (95%) than stomach cramps (82%) or vomiting (64%). The median duration of diarrhea (13 days, interquartile range 4 to 27) exceeded that of vomiting (1 day, interquartile range 1 to 2).

Of the 224 persons who were ill, 70 (31%) were categorized as definite cases (those with a pathogen identified in stool specimen reported by mid-July); 107 (48%) as probable cases (those with no pathogen identified by mid-July but diarrhea lasting 4 or more days); and 47 (21%) as possible cases (those with no pathogen identified by mid-July and diarrhea lasting less than 4 days). Of the definite cases, *Giardia lamblia* was identified in 58. Other pathogens were identified, and some cases had dual infections (Table 1).

Epidemic curves for both diarrhea and vomiting suggested a point-source outbreak with peak onset from 5 to 7 June (Figure 1). Without

Address for correspondence: Rachel Hardie, PHLS, Communicable Disease Surveillance Centre, 61 Colindale Avenue, London NW9 5EQ, United Kingdom; fax: 44-181-200-7868; e-mail: R.Hardie@cdsc.nthames.nhs.uk.

Table 1. Pathogens identified in definite cases

| Pathogen | No. of definite cases[a] | (%) |
|---|---|---|
| *Giardia lamblia* | 58 | (83) |
| *Cryptosporidium parvum* | 11 | (16) |
| *Campylobacter* spp. | 4 | (6) |
| *Salmonella* spp. | 3 | (4) |
| *Entamoeba histolytica* | 2 | (3) |
| Rotavirus | 1 | (1) |

[a]Sum exceeds 70 because of co-infections.

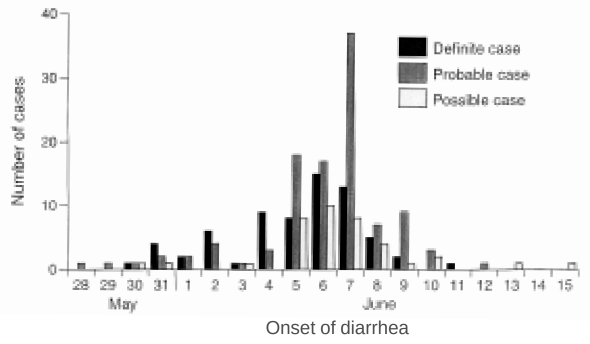

Figure 1. Epidemic curve for diarrhea, by category of case.

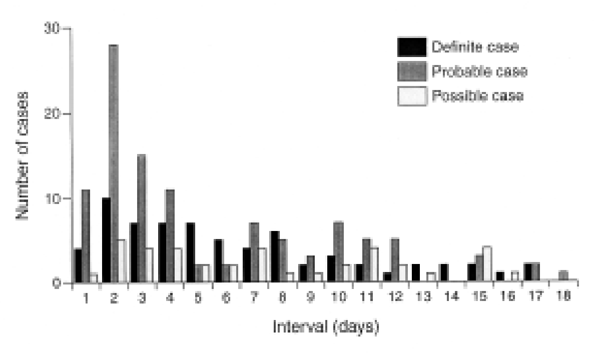

Figure 2. Interval between arrival at hotel and onset of illness (vomiting or diarrhea), by category of case.

knowing the date of exposure, we could not calculate the exact incubation period; instead, we estimated the upper limit of the incubation period by calculating the interval between arrival at the hotel and onset of illness (Figure 2). The short incubation period (most persons became ill within 3 days) and high proportion of cases reporting vomiting at an early stage suggest that some initial illness was caused by a viral gastroenteropathic pathogen. Supporting this hypothesis was the isolation of small round-structured virus (Norwalk virus) from one stool specimen obtained in Greece and of rotavirus in the United Kingdom.

No stool specimens were examined for *G. lamblia* in Greece. During telephone interviews in the United Kingdom, members of 20 groups were advised to provide further stool specimens for testing for *G. lamblia.* By the end of the investigation, at least one stool specimen had been examined for ova, cysts, and parasites for 142 (63%) of the cases. Of the 58 confirmed cases of giardiasis, 51 were treated with metronidazole; 40 cases were treated empirically.

During the telephone interviews, travelers were asked about problems with the food or water in Greece: 54 reports were documented from 51 groups. Of the reports, 32 (59%) identified problems with food (tasted strange, inadequately cooked or heated, left out uncovered); 27 (50%) identified problems with room water (sewage smell and discoloration, principally around 4 June); and none identified problems with drinking water (travelers drank bottled water). However, 7 (13%) identified problems with drinks reconstituted with tap water. None identified problems with swimming pool water. Statistical analysis showed that attendance at children's activities and problems with food were not associated with certainty of diagnosis. However, date of departure, consumption of reconstituted orange juice, consumption of raw vegetables and salads, and reports of problems with water in the hotel room were significantly associated with illness (Table 2).

This outbreak of giardiasis was the largest reported in the United Kingdom since 1985 (1) and the first identified in tourists returning from abroad. The investigation, which relied on active case finding, was complicated by occurrence of illness in more than one country and multiplicity of pathogens identified. Evidence suggests that two principal illnesses, both due to sewage contamination of the water supply, were responsible for the outbreak: an epidemic viral gastroenteritis and giardiasis.

The epidemic curve suggests a point-source outbreak (although some of the viral gastroenteritis may have been transmitted person to person before the peak incidence). If exposure to *G. lamblia* occurred at the same time as that to the pathogen responsible for the viral gastroenteritis, then with its longer incubation period (5

Table 2. Possible risk factors for illness

| Risk factor, chi-square, and p values | Number (%) of guests: Definite cases (n=70, 36 children) yes | no | Probable cases (n=107, 39 children) yes | no | Possible cases (n=47, 21 children) yes | no | Not ill (n=15, 11 children) yes | no |
|---|---|---|---|---|---|---|---|---|
| Departure after 5 June (chi-square=7.3, p=0.007) | 65 (93) | 5 (7) | 99 (93) | 8 (7) | 38 (81) | 9 (19) | 11 (73) | 4 (27) |
| Attendance at children's activities (chi-square=0.01, p=0.92) | 23 (64) | 13 (36) | 25 (64) | 14 (36) | 15 (71) | 6 (29) | 6 (55) | 5 (45) |
| Consumption of raw vegetables and salads (chi-square=3.9, p=0.04) | 52 (74) | 18 (26) | 78 (73) | 29 (27) | 33 (70) | 14 (30) | 6 (40) | 9 (60) |
| Consumption of reconstituted orange juice (chi-square=8.4, p=0.004) | 67 (96) | 3 (4) | 100 (93) | 7 (7) | 43 (91) | 4 (9) | 10 (67) | 5 (33) |
| Problem with water in room (chi-square=7.2, p=0.007) | 49 (70) | 21 (30) | 54 (50) | 53 (50) | 17 (36) | 30 (64) | 9 (60) | 6 (40) |
| Problem with food (chi-square=0.3, p=0.58) | 43 (61) | 27 (39) | 76 (71) | 31 (29) | 26 (55) | 21 (45) | 9 (60) | 6 (40) |

to 25 days vs. 24 to 48 hours for viral gastroenteritis (2), illness due to giardiasis would be expected to merge with recovery from the viral illness. A biphasic epidemic curve would arise with the second peak due to cases in persons who developed symptoms due only to giardiasis.

The following evidence supports the water-borne nature of the outbreak: 1) the epidemic curve suggests a point-source outbreak; 2) several water-borne pathogens were identified in cases (although environmental investigations in Greece at the time of the outbreak found no pathogens, *G. lamblia* was not specifically sought); 3) 129 guests reported that their room water smelled of sewage or was discolored around 4 June, and such reports were more likely from guests in groups with definite cases; 4) consumption of reconstituted orange juice was associated with certainty of diagnosis; 5) although all guests reported avoiding tap water, exposure was almost universal through reconstituted drinks and wet glasses; and 6) the water supply was chlorinated but not filtered, except at certain points within the hotel.

Most nationwide outbreaks of gastrointestinal disease in the United Kingdom, as well as additional cases, are identified through nationally collated laboratory reports. In 1996, of the 5,517 laboratory reports of *G. lamblia* to CDSC from England and Wales, 995 (18%) were associated with foreign travel. This outbreak, however, was initially identified by a local public health epidemiologist; most cases were ascertained largely through the tour operator. Laboratory reports resulted in identification of only 10 cases; a nationwide request for information on related cases resulted in identification of cases from only six groups. Tour operators are likely to collaborate with outbreak investigations, particularly since a European Directive has made them liable for acts and omissions of those with whom they contract to care for their clients (3). In this instance, the tour operator organized an inspection and environmental sampling of the hotel, initiated a survey of food exposure of hotel guests, offered alternative accommodation, informed their clients of the outbreak after their return to the United Kingdom, advised them to obtain an appropriate stool examination, withdrew from assigning clients to the hotel for 1 month while the situation was monitored, and offered guests financial compensation.

Ill travelers may have been more likely than healthy travelers to respond to the tour operator's request to contact CDSC. The number of definite cases, however, was likely to be underrecognized. Guests were classified as having a protozoal illness only if stool specimens were examined specifically for ova, cysts, and parasites; 36% of cases did not have stool specimens examined in this way, and many others had only one stool specimen tested (intermittent excretion of cysts means that the estimated sensitivity of one stool specimen is 50% to 70% [4]). Only half of the laboratories in the Public Health Laboratory Service routinely examine stool specimens for ova, cysts, and parasites (5); most doctors must specifically request the investigation to ensure it is performed.

Given limited health-care resources, the benefits of such an outbreak investigation must be weighed against the costs. In this outbreak, the benefits included identification and appropriate management of cases, as well as provision of information to the tour operator to direct preventative action (which is important, because the investigating body in the country of residence of outbreak patients cannot act directly). Increasing international collaboration in surveillance and investigation of communicable disease outbreaks, particularly within the European Union (EU) (e.g., Enter-Net [6]), may facilitate action by public health professionals in the host country after illness in visitors. The United States/EU Task Force on Communicable Disease is committed to extending Enter-Net beyond Europe, and several countries including the United States, Canada, Australia, South Africa, and Japan are interested in joining.

### Acknowledgments

We acknowledge the information and support provided by consultants in communicable disease control and environmental health officers in the United Kingdom. We thank Mrs. F. Ryan for data entry and the tour operator for collaboration.

Dr. Hardie is a senior registrar in public health medicine at the PHLS Communicable Disease Surveillance Centre in London. In addition to giardiasis, her current research interests include evaluation of health services for tuberculosis and strategies for prevention and control of hepatitis C and methicillin-resistant *Staphylococcus aureus*.

### References

1. Jephcott AE, Begg NT, Baker IA. Outbreak of giardiasis associated with mains water in the United Kingdom. Lancet 1985;29:730-2.
2. Benenson AS, editor. Control of communicable diseases manual. 16th ed. Washington: American Public Health Association; 1995.
3. European Community Council. Council on package travel, package holidays and package tours. Official Journal of the European Communities, 23 Jun 1990. Directive 90/314/EEC.
4. Hill D. Giardiasis. Issues in diagnosis and management. Inf Dis Clin North Am 1993;7:503-25.
5. Pugh C, Morris I. Giardiasis: the epidemiological and resource implications of standardisation. PHLS Microbiology Digest 1997;14:100-1.
6. Yang S. FoodNet and Enter-net: emerging surveillance programs for foodborne diseases. Emerg Infect Dis 1998;4:457-8.

